# Case Report: a rare case of small cell carcinoma of the prostate

**DOI:** 10.3389/fonc.2026.1796935

**Published:** 2026-06-08

**Authors:** Kexin Geng, Feng Gao

**Affiliations:** 1 Hebei Medical University Third Hospital, Shijiazhuang, Hebei, China; 2 Department of Pathology, Hebei Medical University Third Hospital, Shijiazhuang, China

**Keywords:** case report, immunohistochemistry, neuroendocrine prostate cancer, prostate, prostate cancer, small cell carcinoma

## Abstract

Small cell carcinoma of the prostate is an uncommon malignancy with an aggressive clinical course. Its non-specific symptoms and frequently normal prostate-specific antigen (PSA) levels can make early diagnosis difficult. We report a 74-year-old man with progressive defecation difficulty and prostate enlargement. Although his serum PSA was within the normal range, imaging revealed a prostate mass suggestive of local invasion. Needle biopsy established the diagnosis of small cell carcinoma with neuroendocrine marker expression. He later received etoposide plus cisplatin chemotherapy but deteriorated soon after treatment began and died despite resuscitation. This case underscores the importance of early biopsy when imaging suggests an invasive prostate mass, even if PSA is not elevated.

## Introduction

Prostate cancer is common in older men and is predominantly adenocarcinoma. In contrast, small cell carcinoma of the prostate (SCCP) is rare, accounting for approximately 0.5%–2% of prostate cancers, and it typically follows an aggressive course with early invasion and poor outcomes (1). Because symptoms are often non-specific and serum prostate-specific antigen (PSA) may remain within the normal range, SCCP can be difficult to recognize before advanced disease is evident. Evidence on its diagnosis and treatment remains limited, and management is mainly based on small case series and experience from other neuroendocrine carcinomas. The clinical and radiological findings that should prompt early biopsy in patients with invasive-appearing prostate lesions but non-elevated PSA levels remain unclear. We report a case of SCCP presenting with progressive defecation difficulty, normal PSA levels, and imaging findings suspicious for local invasion. This case highlights the need for early biopsy when clinical or radiological findings suggest aggressive prostate disease despite non-elevated PSA levels.

## Case description

A 74-year-old man presented with difficulty defecating, and physical examination revealed an enlarged prostate. He had undergone a routine check-up 18 days before admission, which noted prostate enlargement, and he subsequently developed progressive defecatory difficulty. Digital rectal examination showed a markedly enlarged and firm prostate with an obscured central groove; no discrete nodules were palpated, and there was no tenderness. He was admitted with a provisional diagnosis of benign prostatic hyperplasia. The patient had a history of bladder cancer and had undergone transurethral resection of a bladder tumor at our hospital 3 years earlier; pathology showed high-grade papillary urothelial carcinoma, followed by intravesical chemotherapy. On admission, his general condition was fair without obvious cachexia.

### Diagnostic assessment

**Figure 1:**
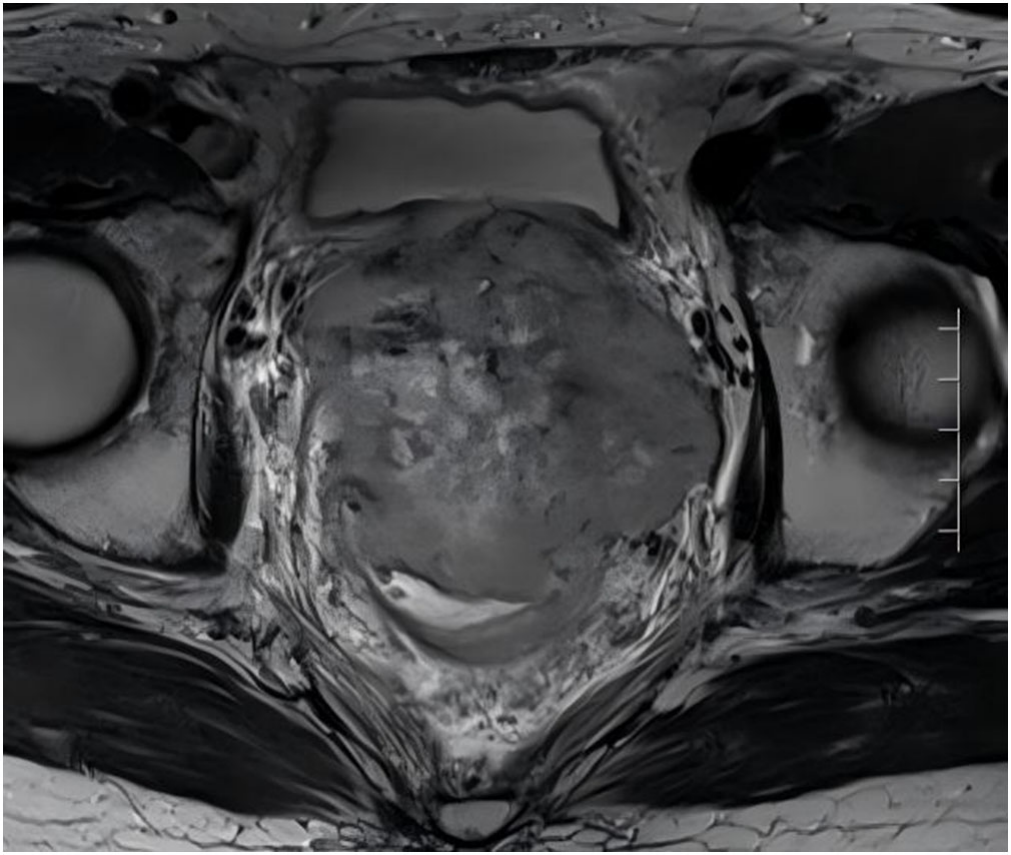
Prostate MRI (3.0T). The prostate is enlarged with an ill-defined lesion.

**Figure 2:**
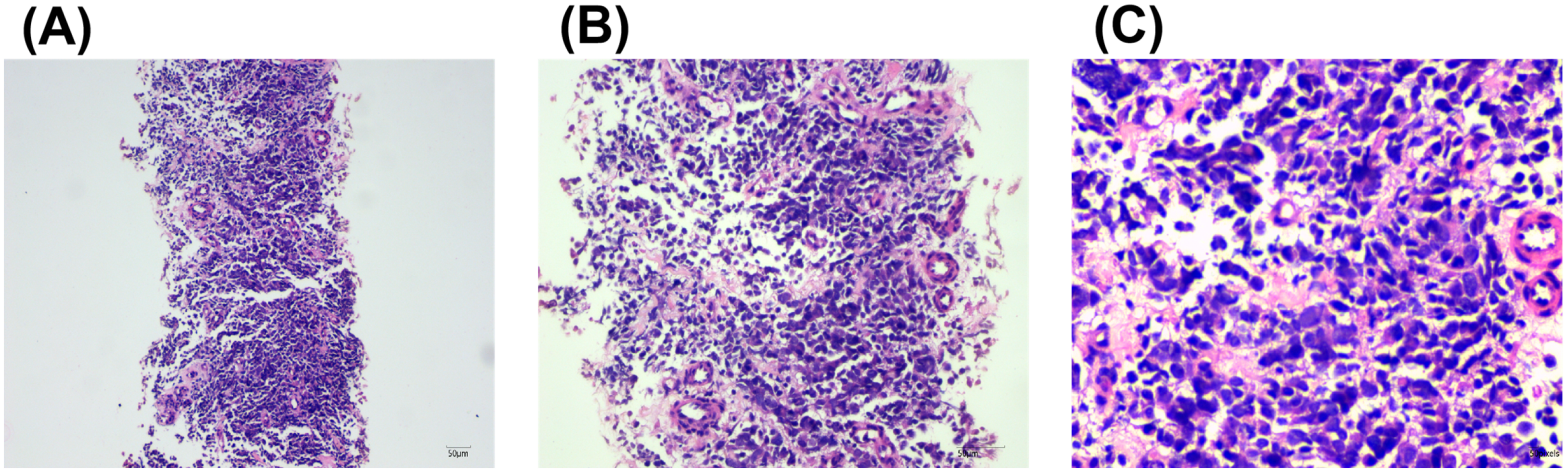
Hematoxylin and eosin (H&E)-stained sections of the prostate needle biopsy. **(A)** Tumor cells show a diffuse sheet-like growth pattern (×100). **(B)** The tumor is composed predominantly of small cells with scant cytoplasm and hyperchromatic nuclei (×200). **(C)** High-power view shows densely packed tumor cells with a high nuclear-to-cytoplasmic ratio (×400).

**Figure 3:**
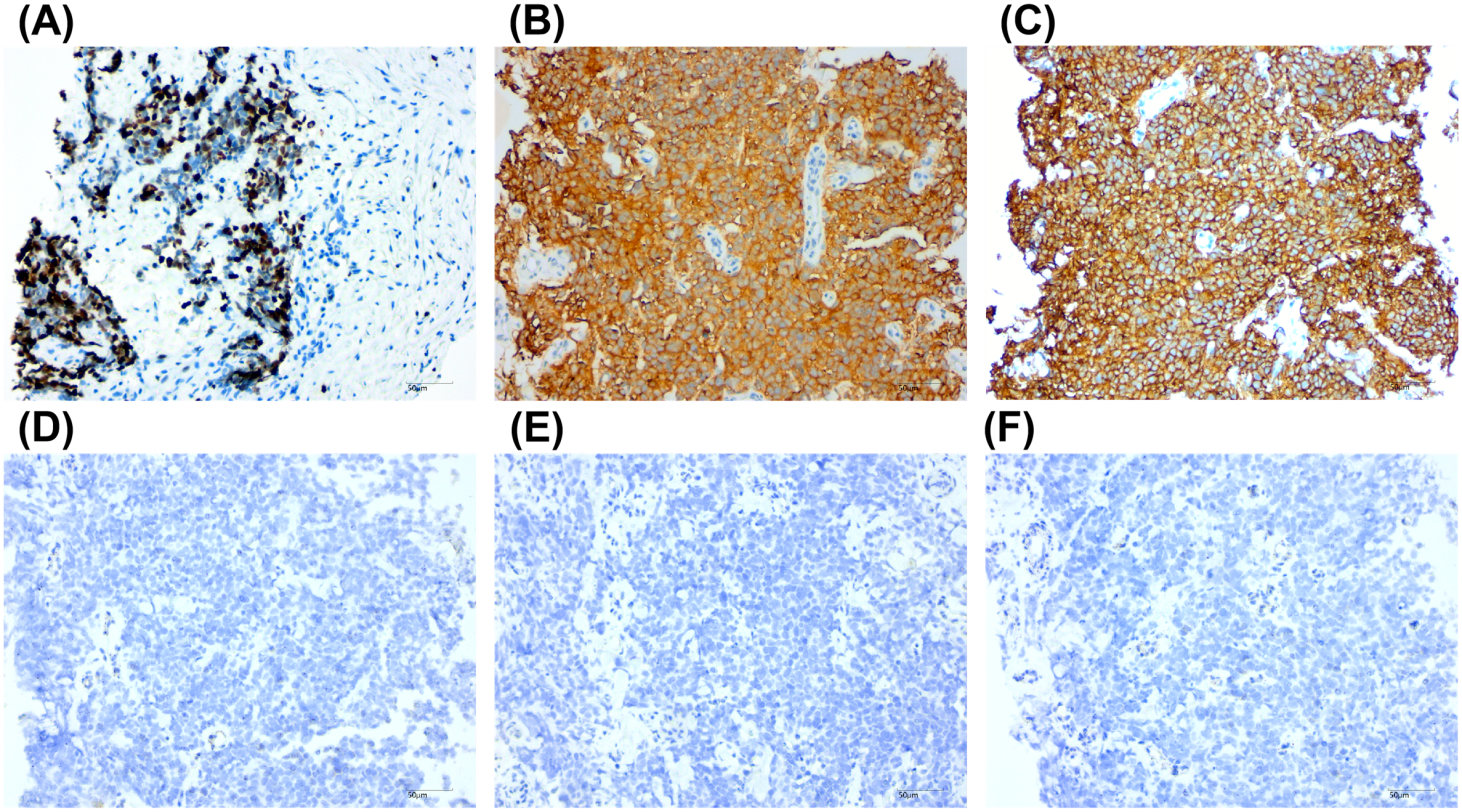
Immunohistochemical staining of the prostate needle biopsy. **(A)** Ki-67 shows an increased labeling index in tumor cells (~50%) (×200). **(B)** CD56 shows diffuse membranous and cytoplasmic positivity in tumor cells (×200). **(C)** Synaptophysin shows diffuse granular cytoplasmic positivity in tumor cells (×200). **(D)** CK5/6 is negative in tumor cells (×200). **(E)** P504S is negative in tumor cells (×200). **(F)** p63 is negative in tumor cells (×200).

Serum total PSA (tPSA) was 1.390 ng/mL, free PSA (fPSA) was 0.276 ng/mL, and the PSA level was not elevated. Color Doppler ultrasound demonstrated diffuse prostate enlargement with irregular margins (69 × 78 × 63 mm). Prostate MRI (3.0T) showed a lesion suspicious for malignancy, with suspected seminal vesicle involvement and a right iliac perivascular lymph node metastasis; invasion of the anterior rectal wall could not be excluded (**Figure 1**). A prostate biopsy was performed on hospital day 6. Histology showed small cell carcinoma with necrosis. The biopsy demonstrated diffuse sheet-like and nested infiltrative growth of a densely cellular tumor with scant stroma. Tumor cells were mainly small to intermediate in size, with scant cytoplasm, a high nuclear-to-cytoplasmic ratio, and indistinct borders. Nuclei were round to oval and hyperchromatic, with coarsely granular chromatin and inconspicuous nucleoli. Focally, eosinophilic amorphous material with nuclear debris was present (**Figure 2**). Immunohistochemistry was positive for CD56, synaptophysin (Syn), and pan-cytokeratin, with a Ki-67 labeling index of approximately 50%. P504S, p63, CK5/6, CK34βE12, CK7, and p40 were negative, and CAM5.2 showed weak staining (**Figure 3**). The main differential diagnoses were poorly differentiated prostatic adenocarcinoma, lymphoma, and metastatic small cell carcinoma from another primary site. Poorly differentiated adenocarcinoma was less favored because the tumor was negative for P504S and expressed neuroendocrine markers. Lymphoma was also not favored, given the pan-cytokeratin positivity and the absence of typical lymphoid morphology. In addition, the available clinical and imaging findings did not identify an extra-prostatic primary tumor, which supported a primary prostatic origin. An outside consultation reported a higher Ki-67 labeling index of approximately 90%. Overall, the findings supported small cell carcinoma of the prostate. A whole-body bone scan showed no definite evidence of metastasis.

### Therapeutic intervention

On hospital day 12, the patient and his family decided to pursue transfer and requested discharge. He was admitted to another hospital approximately 2 weeks later, where cytokeratin 19 fragment antigen 21-1 (CYFRA 21–1) was 5.19 ng/mL, and neuron-specific enolase (NSE) was 191.00 ng/mL (both above the upper limit of normal). Chemotherapy was initiated 2 days after admission using an EP regimen: etoposide (0.1 g) and cisplatin (20 mg) were infused intravenously on days 1–4, with diphenhydramine and dexamethasone as premedication and dolasetron for antiemetic prophylaxis.

### Follow-up and outcomes

On chemotherapy day 4, he developed an acute change in mental status with high fever (38.5 °C–39.3 °C). Laboratory testing showed marked leukocytosis [white blood cell count (WBC) 35.85 × 10^9^/L] with neutrophil predominance (86.2%), markedly elevated inflammatory markers [C-reactive protein (CRP) > 200 mg/L; procalcitonin (PCT) 34.57 ng/mL], anemia [hemoglobin (Hb) 96 g/L], and increased red cell distribution width (RDW). Hematology considered acute hemolysis and a leukemoid reaction, and infectious or autoimmune etiologies were not excluded. The patient progressed to cardiopulmonary failure within approximately 2 hours of symptom onset and was pronounced dead despite resuscitative efforts. The patient’s clinical course is summarized in **Table 1**.

**Table 1:** Timeline of the patient’s clinical course.

Time point	Key events	Findings/outcomes
3 years before admission	Transurethral resection of bladder tumor (TURBT); intravesical chemotherapy	High-grade papillary urothelial carcinoma
18 days before admission	Routine check-up	Prostate enlargement noted
Pre-admission	Symptom evolution	Progressive difficulty defecating
HD 0	Admission; digital rectal examination (DRE)	Markedly enlarged, firm prostate; central sulcus effaced; no discrete nodules; non-tender; initial impression: benign prostatic hyperplasia (BPH)
HD 0–5	PSA and imaging	PSA not elevated. Ultrasound: diffuse enlargement with irregular margins. MRI: lesion concerning for malignancy with suspected seminal vesicle involvement; pelvic node and possible anterior rectal wall involvement
HD 6	Prostate biopsy	Small cell carcinoma with necrosis
HD 7	Immunohistochemistry (IHC); outside review; bone scan	CD56/Syn/pan-CK positive; Ki-67 ~50% in our staining and ~90% in outside consultation; prostate/basal markers negative; bone scan showed no definite osseous metastasis
HD 12	Discharge for transfer	Family requested transfer of care
~2 weeks later	Re-admission elsewhere	CYFRA 21–1 and NSE elevated
HD 2 at receiving hospital	EP chemotherapy	Etoposide plus cisplatin administered
Day 4 of EP	Abrupt decline	High fever and altered mental status with marked inflammatory response; rapid cardiopulmonary collapse; death despite resuscitation

HD, hospital day of index admission; PSA, prostate-specific antigen; TURBT, transurethral resection of bladder tumor; DRE, digital rectal examination; BPH, benign prostatic hyperplasia; IHC, immunohistochemistry.

## Discussion

Pathological examination in this patient supported a diagnosis of SCCP, one of the most aggressive forms of neuroendocrine prostate cancer (NEPC). SCCP is rare, but it is the most common neuroendocrine carcinoma of the prostate (2). It may arise *de novo* or develop from pre-existing acinar adenocarcinoma, with the latter reported more often. Treatment-emergent SCCP usually occurs after androgen deprivation therapy or more potent androgen receptor pathway inhibition, particularly in metastatic castration-resistant disease (3). In that setting, endocrine therapy alone is often insufficient. Several clinical clues may prompt biopsy, including rapid tumor progression, osteolytic bone metastases, low PSA despite a heavy tumor burden, and increased neuroendocrine serum markers (1, 4). In this patient, there was no known history of prostate cancer or endocrine therapy, so primary SCCP at initial diagnosis was favored. The clinical course, imaging findings, and pathology were compatible with this diagnosis. However, because the diagnosis was based on needle biopsy, a mixed tumor or focal adenocarcinoma component cannot be completely excluded.

SCCP mainly affects older men and often presents with non-specific symptoms. Early disease may be clinically silent, whereas symptoms usually become evident when the tumor grows rapidly or invades adjacent structures. Patients may develop progressive lower urinary tract obstruction, pelvic or perineal discomfort, or symptoms caused by rectal compression, such as difficulty defecating and rectal bleeding. At diagnosis, SCCP is often locally advanced or metastatic. Approximately 60% of patients have distant metastases at presentation, with reported sites including the lung, brain, liver, and bone (1). In our patient, progressive defecation difficulty was the main complaint. Digital rectal examination and imaging showed a prostatic mass with local invasion, but serum PSA showed no clear elevation. This “high tumor burden, low PSA” pattern differs from typical acinar adenocarcinoma, which more often shows PSA elevation and a slower clinical course (1). PSA may still rise in some SCCP cases, particularly in mixed tumors or in tumors retaining partial adenocarcinoma differentiation. In such cases, the PSA increase is likely driven by the adenocarcinoma component. This distinction is clinically useful because a low PSA level should not delay biopsy when symptoms or imaging suggest aggressive prostate disease.

Imaging findings in SCCP are not specific. The tumor may appear as diffuse prostate enlargement or an irregular mass with ill-defined margins, and extra-prostatic extension is common. Seminal vesicle involvement, anterior rectal wall invasion, and pelvic lymph node metastasis may also be suspected on imaging (5). These findings overlap with poorly differentiated adenocarcinoma, so imaging alone rarely establishes the diagnosis. Histopathological confirmation remains essential. On H&E staining, SCCP typically consists of small, poorly differentiated cells with scant cytoplasm, a high nuclear-to-cytoplasmic ratio, indistinct borders, hyperchromatic nuclei, fine granular chromatin, inconspicuous nucleoli, nuclear molding, necrosis, and brisk mitotic activity. Gland formation is usually absent (1) (6). These features resemble small cell carcinoma of the lung, and the histological description often follows the same morphological criteria. In small biopsy specimens, crush artifact, necrosis, and limited tissue may obscure architecture and nuclear detail. In this setting, SCCP can mimic other small round cell tumors, including Gleason grade 5 poorly differentiated acinar adenocarcinoma and lymphoma.

Immunohistochemistry is important for confirming lineage and narrowing the differential diagnosis. In our case, tumor cells were positive for pan-cytokeratin, Syn, and CD56, supporting an epithelial neuroendocrine carcinoma. Cytokeratin positivity made lymphoma less likely, although lymphoid markers should be added if cytokeratin staining is absent or equivocal (6, 7). SCCP typically expresses neuroendocrine markers, such as Syn, CD56, and/or chromogranin A, and in some cases may also express TTF-1. In contrast, conventional prostate adenocarcinoma markers, including PSA, prostate-specific acid phosphatase (PSAP), P504S/alpha-methylacyl-CoA racemase (P504S/AMACR), and androgen receptor (AR), are often negative or only focally weak. This immunophenotype helps distinguish SCCP from poorly differentiated acinar adenocarcinoma. In this case, CD56 and Syn were positive, whereas P504S, p63, CK5/6, and CK34βE12 were negative. These findings supported small cell neuroendocrine differentiation and did not support basal or squamous differentiation. The negative P504S result further favored a pure small cell carcinoma pattern, although the possibility of an unsampled adenocarcinoma component cannot be fully excluded because only biopsy material was available. The reported Ki-67 labeling index differed between the outside consultation and our subsequent staining. The outside consultation estimated Ki-67 at approximately 90%. After the diagnostic block had been loaned out, the remaining sections from that block were exhausted, and repeat staining on the same material was not possible. We therefore performed Ki-67 staining on another tumor block from a different area, which showed a labeling index of approximately 50%. This difference may reflect intratumoral heterogeneity, sampling from different tumor foci, variation in tumor cellularity, or differences in scoring. Nevertheless, both results indicate a highly proliferative tumor.

Systemic treatment for SCCP is largely adapted from small cell lung cancer practice. A platinum agent plus etoposide is the most common first-line regimen, including etoposide plus cisplatin or etoposide plus carboplatin (3) (8),. Retrospective studies and reviews have reported objective responses, but these responses are often short-lived, and the overall prognosis remains poor. Most patients survive approximately 1 year after diagnosis (3, 9). Newer approaches, including combinations with immunotherapy or targeted agents, are being explored, but most data remain limited to trials and early reports. For example, immunotherapy combined with platinum–etoposide has been investigated in extrapulmonary neuroendocrine carcinoma or NEPC, but the magnitude of benefit and the patients most likely to benefit remain unclear (10). In the present case, the patient died on day 4 after initiation of EP chemotherapy following an abrupt terminal event. Given the limited available data, this timing alone should not be interpreted as evidence of treatment failure or treatment-related mortality. Emergency records documented high-grade fever, marked neutrophilic leukocytosis, and striking elevations of CRP and PCT. These findings are consistent with a severe systemic inflammatory response and raise concern for infection, including possible sepsis. The hematology team also considered acute hemolysis and a leukemoid reaction, while infectious and autoimmune etiologies were not excluded. Because key terminal laboratory and etiologic data were unavailable, the precise cause of death cannot be determined. We therefore describe the terminal course without assigning a definitive cause. Nonetheless, this case illustrates the aggressive and clinically unstable course of SCCP. After systemic therapy is initiated, close monitoring and early diagnostic workup for infection, hematologic derangements, and acute organ dysfunction are warranted.

### Patient perspective

The family felt the condition was already severe at first detection. They reported that the patient repeatedly declined interventions for defecation difficulty.

## Conclusion

Small cell carcinoma of the prostate is rare and aggressive, and PSA can stay low even with invasive disease. An early biopsy is warranted when imaging shows an invasive prostatic mass with a low PSA. Immunohistochemistry supports the diagnosis and helps avoid delay.

## Data Availability

The raw data supporting the conclusions of this article will be made available by the authors, without undue reservation.
