# Supplementary material for: Case Report: a rare case of small cell carcinoma of the prostate
Source: Front Oncol. 2026 Jun 8;16:1796935. doi: 10.3389/fonc.2026.1796935 (PMC13283838; doi:10.3389/fonc.2026.1796935)
Supplement: Supplementary file 1 [file DataSheet1.pdf]

CARE case report guidelines

CARE Checklist of information to include when writing a case report

(cc) BY-NC-ND

Topic	Item	Checklist item description	Reported location in manuscript
Title	1	The diagnosis or intervention of primary focus followed by the words "case report"	Title page
Key Words	2	2 to 5 key words that identify diagnoses or interventions in this case report, including "case report"	Keywords section
Abstract (no references)	3a	Introduction: What is unique about this case and what does it add to the scientific literature?	Abstract
	3b	Main symptoms and/or important clinical findings	Abstract
	3c	The main diagnoses, therapeutic interventions, and outcomes	Abstract
	3d	Conclusion—What is the main "take-away" lesson(s) from this case?	Abstract
Introduction	4	One or two paragraphs summarizing why this case is unique (**may include references**)	Introduction
Patient Information	5a	De-identified patient specific information.	Case description
	5b	Primary concerns and symptoms of the patient.	Case description
	5c	Medical, family, and psycho-social history including relevant genetic information	Case description
	5d	Relevant past interventions with outcomes	Case description
Clinical Findings	6	Describe significant physical examination (PE) and important clinical findings.	Case description
Timeline	7	Historical and current information from this episode of care organized as a timeline	Timeline; Table 1
Diagnostic Assessment	8a	Diagnostic testing (such as PE, laboratory testing, imaging, surveys).	Diagnostic assessment
	8b	Diagnostic challenges (such as access to testing, financial, or cultural)	Not Applicable
	8c	Diagnosis (including other diagnoses considered)	Diagnostic assessment
	8d	Prognosis (such as staging in oncology) where applicable	Diagnostic assessment
Therapeutic Intervention	9a	Types of therapeutic intervention (such as pharmacologic, surgical, preventive, self-care)	Therapeutic intervention
	9b	Administration of therapeutic intervention (such as dosage, strength, duration)	Therapeutic intervention
	9c	Changes in therapeutic intervention (with rationale)	Not Applicable
Follow-up and Outcomes	10a	Clinician and patient-assessed outcomes (if available)	Follow-up and outcomes
	10b	Important follow-up diagnostic and other test results	Follow-up and outcomes
	10c	ntervention adherence and tolerability (How was this assessed?)	Not Applicable
	10d	Adverse and unanticipated events	Follow-up and outcomes
Discussion	11a	A scientific discussion of the strengths AND limitations associated with this case report	Discussion
	11b	Discussion of the relevant medical literature **with references**.	Discussion
	11c	The scientific rationale for any conclusions (including assessment of possible causes)	Discussion
	11d	The primary "take-away" lessons of this case report (without references) in a one paragraph conclusion	Conclusion
Patient Perspective	12	The patient should share their perspective in one to two paragraphs on the treatment(s) they received	Patient consent
Informed Consent	13	Did the patient give informed consent? Please provide if requested	Yes ☑ No ☐
